# From Solution Studies of Pharmaceuticals (Aspirin and Related Compounds) to the Thermodynamics of Aspirin-β-Cyclodextrin Interaction in water and N,N-Dimethylformamide

**DOI:** 10.3390/ijms231911750

**Published:** 2022-10-04

**Authors:** Angela F. Danil de Namor, Alexandros Cambanis, Nawal Al Hakawati, Rasha Khalife

**Affiliations:** 1 Laboratory of Thermochemistry, Department of Chemistry, University of Surrey, Guildford, Surrey GU2 7XH, UK; 2 Department of Biological Sciences, Faculty of Science, Beirut Arab University, Tripoli P.O. Box 11-5020, Lebanon; 3 School of Chemical, Biological and Environmental Engineering, Oregon State University, 116 Johnson Hall 105 SW 26th Sr, Corvallis, OR 97331, USA

**Keywords:** aspirin, β-cyclodextrin, thermodynamic parameters

## Abstract

The solution behavior of pharmaceuticals (acetylsalicylic acid, 4-acetoxybenzoic acid and 5-acetylsalicylic acid) in water and N,N-Dimethylformamide (DMF) at 298.15 K were investigated through solubility, conductance and calorimetric measurements. Taking into account the formation of ion pairs of these pharmaceuticals in water, the solution Gibbs energies of the dissociated electrolytes in this solvent were calculated. Thus, the solution thermodynamics of these compounds in water are reported using enthalpy data obtained by calorimetry. These pharmaceuticals undergo solvation when exposed to a saturated atmosphere of DMF. As the composition of the solid is not the same as that in solution, the Gibbs energy of the solutions of these compounds could not be obtained; only enthalpy data are reported. The thermodynamics of the interaction of acetylsalicylic acid (aspirin) with β-cyclodextrin in water and DMF is fully discussed, emphasizing the two different processes that take place in water at the two different pHs. In all cases, the favorable Gibbs energies for these processes are entropically controlled, mainly resulting from the higher dehydration/desolvation that the receptor undergoes upon interaction with the guest.

## 1. Introduction

Pharmaceutical industries are facing many drawbacks due to the difficulties encountered in the administration of several drugs, such as their low solubility, drug delivery, instability and low bioavailability [1,2,3,4,5].

Among the most commonly used drugs is acetylsalicylic acid, obtained by Hoffman [6], which was named aspirin and is known for its antithrombotic effects [7]. It is recommended that all patients with a history of heart problems or strokes take aspirin regularly as a prophylactic measure. On the other hand, aspirin can have serious side effects and in excess amounts can be fatal, as its toxicity is directly proportional to the dose. Among these effects are liver toxicity as well as asthma and allergic diseases [8]. Because of these side effects, Rainsford [9] suggested the development of inclusion complexes that are able to mask the direct interaction between the drug and the mucosal cells to avoid or at least attenuate the side effects of the drug using cyclodextrins as receptors. Further advantages of choosing cyclodextrins are their nontoxicity, given that these are essentially polymers of glucose, and that the solubility increase of the drug–cyclodextrin complex relative to the drug leads to a positive impact on the bioavailability of the drug [10,11,12]. There have been a number of reports on the solubility of drugs, including aspirin, in water and nonaqueous solvents as well as attempts to thermodynamically characterize the solution properties of aspirin and other drugs and their complexation with cyclodextrins [13,14,15,16,17,18,19,20].

**Figure 1:**
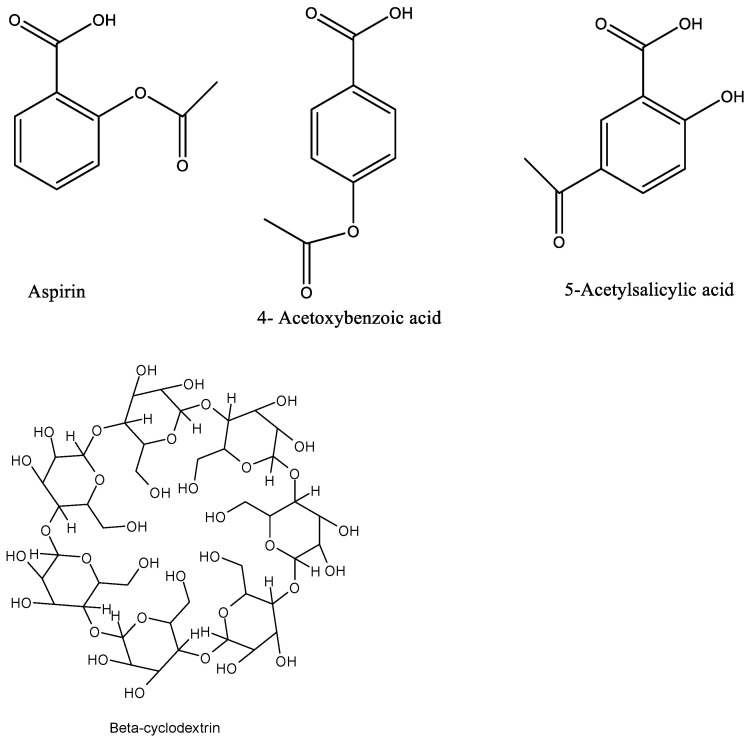
Chemical structures of acetylsalicylic acid (aspirin), 4-acetoxybenzoic acid, 5-acetylsalicylic acid and β-cyclodextrin.

Although several reports were found in the literature related to the interactions of aspirin and related compounds with cyclodextrins due to the need to overcome the side effects of the former on certain patients, little is known about the solution properties of these compounds. It is well-established that the solvation of the reactants and the product plays a key role in complexation processes, and these have been extensively discussed previously by Danil de Namor and coworkers [21,22,23]. Thus, in this paper we report the solution thermodynamic parameters of acetylsalicylic acid (aspirin) and related compounds (Figure 1) in water and DMF at 298.15 K and the thermodynamics of the complexation of acetylsalicylic acid (aspirin) and beta-cyclodextrin in these solvents.

## 2. Results and Discussion

### 2.1. Solubility Measurements

The solubility data for acetylsalicylic acid, 4-acetoxybenzoic acid and 5-acetylsalicylic acid in water at 298.15 K are reported in Table 1. The data for acetylsalicylic acid show reasonable agreement with those reported in the literature (2.32 × 10^−^^2^ mol/Kg or 2.31 × 10^−2^ mol dm^−3^) at 299.65 K [16]. Taking into account the density of water at this temperature, it follows that the solubility of acetylsalicylic acid at this temperature on the molar scale is quite close to the value reported in Table 1 at 298.15 K and slightly differs from the value of 1.78 × 10^−2^ mol/dm^−3^ [17]. Solvate formation was tested in water but was not detected. 

As far as DMF is concerned, these compounds undergo solvate formation when exposed to a saturated atmosphere of this solvent. Therefore, quantitative data on their solubilities could not be obtained in this solvent. Given that acetylsalicylic acid and related compounds are weak acids in water, ions and ion pairs will be present in water. Therefore, in order to calculate the standard Gibbs energy for the fully dissociated acid, conductance measurements were performed, and these are discussed below.

### 2.2. Conductance Measurements

Conductance Measurements of Acetylsalicylic Acid, 4-Acetoxybenzoic Acid and 5-Acetylsalicylic Acid in Water at 298.15 K

**Figure 2:**
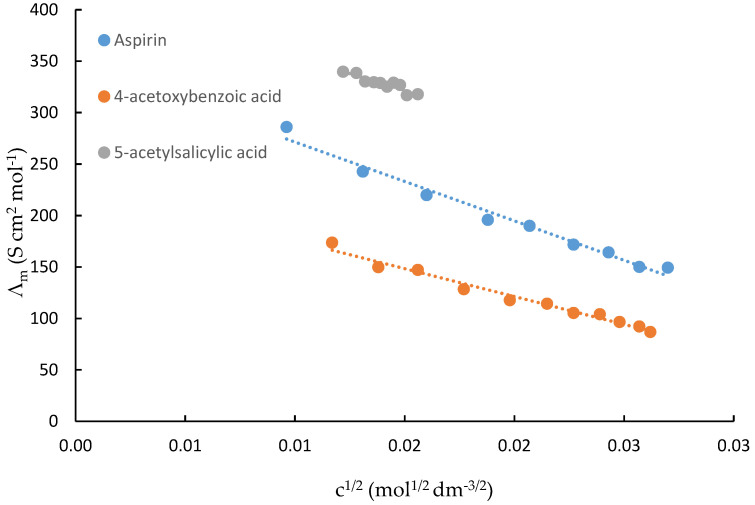
Conductance measurements of acetylsalicylic acid (aspirin), 4-acetoxybenzoic acid and 5-acetylsalicylic acid in water at 298.15 K.

Plots of the molar conductance (Λ_m_) against the c^1/2^ values of acetylsalicylic acid, 4-acetoxybenzoic acid and 5-acetylsalicylic acid in water are shown in Figure 2. A computer program was used to calculate the molar conductance at an infinite dilution, Λ^0^_m_, and the association or ion-pair formation constant (K_ass_) for the process represented by Equation (1), and the molar conductance, Λm (S cm^2^ mol^−1^), was calculated by the Fuoss–Hsia equation using the expansion suggested by Fernandez Prini as described elsewhere [24]. Table 2 lists the values for Λ^0^_m_, the ion-pair formation constant, and K_ip_ (Equation (2)) as well as the dissociation constant, K_a_, and the pK_a_ values of acetylsalicylic acid, 4-acetoxybenzoic acid and 5-acetylsalicylic acid in water at 298.15K.

H^+^ (aq) + A^−^ (aq) ⇌ HA (aq) (1)

The ion-pair formation constant for the process described by Equation (1) is expressed in terms of activities, *a,* as shown in in Equation (2):

(2) $$K_{\mathrm{ip}}=\frac{a_{HA}}{a_{H^{+}}*a_{A^{-}}}=\frac{\left[HA\right]*\gamma_{HA}}{\left[H^{+}\right]*[A^{-}]*\gamma_{\pm i}{}^{2}}$$

In Equation (2), concentrations are in mol dm^−3^, and γ ± *i^2^* is the mean molar ionic coefficient calculated by the use of the Debye–Hückel equation, where γ_HA_ is the activity coefficient of the un-ionized species. In very dilute solutions, γ_HA_ may be considered equal to unity. As far as the standard Gibbs energy of the solution in the standard state of 1 mol dm^−3^, ∆_s_G^0^, is concerned, the application of Equation (3) requires knowledge of the ion-pair formation constant in solution. The availability of K_ip_ and solubilities in water allows the calculation the ionic molar concentration, c_i_, and the molar concentration of ion pairs, c_ip_, as previously shown [24]. The data refer to the standard state (1 mol dm^−3^).

Δ_s_G^0^= −RTln (c_i_^2^ * γ±_i_^2^) (3)

Based on data from Pierre and Jencks [25], it was estimated that, at the end of the conductance measurements of acetylsalicylic acid, 5% of it was hydrolyzed. It is assumed that their percentage would not have significantly affected the obtained Λ^0^_m_, K_ip_, K_a_ and pK_a_ values. In the case of 4-acetoxybenzoic acid, this percentage was estimated to be even lower. The availability of Λ^0^_m_ leads to the calculation of the ionic conductances at an infinite dilution for the anion (λ_A_^−^) since the value for the proton (λ_H_^+^ = 349.8 S cm^2^ mol^−^^1^) [26] is known. For this purpose, the law of the independent migration of ions (Equation (4)) is used:

Λ^0^_m_ = υ_+_ ∗ λ_H_^+^ + υ_−_ ∗ λ_A_^−^ (4)

In this equation, υ_+_ is the number of cations and υ_−_ is the number of anions.

Thus, values of 42.2, 51.2 and 39.2 S cm^2^ mol^−1^ were calculated, respectively, for the ionic conductivities at an infinite dilution in water at 298.15 K for acetylsalicylate, acetoxybenzoate and 5-acetylsalicylate anions. As far as the salicylate anions are concerned, their λ_A_ values, as expected, were higher than that for the unsubstituted anion (λ_A = _ 36.14 S cm^2^ mol^−1^) [27]. A similar pattern was found for the substituted benzoate anion relative to the unsubstituted one (λ_A =_ 32.2 S cm^2^ mol^−1^) [28]. On the other hand, the 4-acetoxybenzoic acid anion had a higher increase in the λ^−^ value than the other two anions, which may be due to the lower solvation of this anion relative to the others.

As far as the pKa values are concerned, the data in Table 2 show that, among the considered systems, 4-acetoxybenzoic acid had the highest pK_a_ value (4.34, which is in excellent agreement with the value of 4.38 [29] reported in water at this temperature and determined by conductimetry). Hence, it is the weakest acid, and therefore, its conjugate base has a greater proton affinity than acetylsalicylic acid and 5-acetylsalicylic acid. For acetylsalicylic acid, the pKa value shown in Table 2 is in fair agreement with the values of 3.483 [30], 3.49 [30,31] and 3.50 [32,33,34,35] reported in water at this temperature. 

5-acetylsalicylic acid showed the lowest pKa value (3.04) among this series. Consequently, it was more acidic than acetylsalicylic acid and 4-acetoxybenzoic acid. This can be explained on structural terms, given that the acetyl group at position 5 of the benzene ring is an electron-withdrawing group (deactivating group), stabilizing this anion in water and making it a stronger acid.

**Figure 3:**
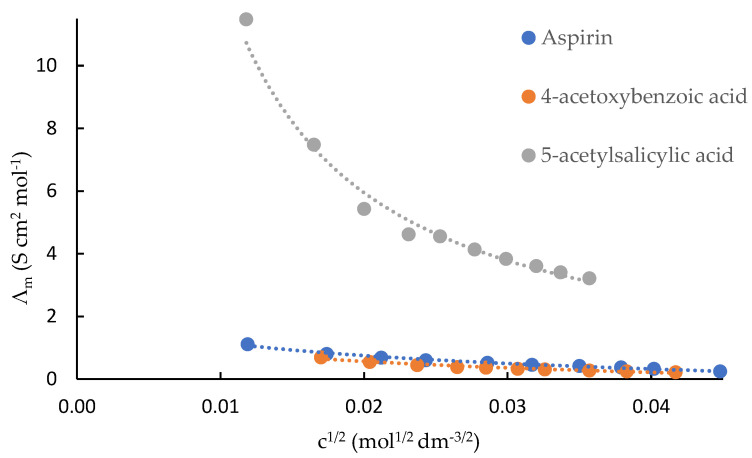
Conductance measurements of acetylsalicylic acid (aspirin), 4-acetoxybenzoic acid and 5-acetylsalicylic acid in DMF at 298.15 K.

Plots of molar conductances (Λ_m_) against the c^1/2^ of acetylsalicylic acid, 4-acetoxybenzoic acid and 5-acetylsalicylic acid in DMF are shown in Figure 3. The Shed turbo basic program was unable to derive accurate data of these compounds in this solvent. Hence, K_ip_ values were not calculated. For the three compounds in DMF, ion pairs predominated, and this is clearly reflected in their extremely low Λ_m_ values.

Given that most enthalpy data reported in the literature were obtained by the use of the van ’t Hoff equation, which has well-established limitations [22,36,37], we proceeded with the determination of solution enthalpies by calorimetry, as discussed below.

### 2.3. Standard Enthalpies of Solution of Acetylsalicylic Acid, 4-Acetoxybenzoic Acid and 5-Acetylsalicylic Acid in Water and DMF at 298.15 K

The enthalpies of solution of acetylsalicylic acid in water at various concentrations are reported in Table 3. No measurements could be carried out at higher concentrations since the dissolution rate of this compound in water was relatively slow. Therefore, the higher reported concentration is the one at which acetylsalicylic acid is fully dissolved in water. Since there were no systematic changes in the Δ_s_H values with changes in the concentration of solute, the standard Δ_s_H^0^ value reported in Table 3 is the average value. The enthalpies of solution of 4-acetoxybenzoic acid and 5-acetylsalicylic acid in water at 298.15 K are also reported in Table 3. In the dissolution of a solute in water or any other solvent, there are two processes involved, as described in Equation (5):

Δ_s_H^0^ = Δ_cl_H^0^ + Δ_solv_H^0^ (5)

In this equation, Δ_cl_H^0^ is the notation used to indicate the crystal lattice process (endothermic), while Δ_solv_H^0^ is the solvation process (exothermic). The results shown in Table 3 for acetylsalicylic acid and related compounds clearly indicate that their dissolution is endothermic (enthalpies of solution are positive), and therefore, the contribution of the crystal lattice enthalpy to the Δ_s_H^0^ values predominates. Thus, the endothermic character of the reaction follows the sequence:

5-acetylsalicylic acid > 4-acetoxybenzoic acid > acetylsalicylic acid

As expected, the Δ_s_H^0^ value for acetylsalicylic acid differs significantly from the apparent value reported by Apelblat and coworkers [16], which was derived from the van ’t Hoff equation, of 22.9 kJ mol^−1^.

### 2.4. Determination of the Gibbs Free Energy and Entropies of Solution of Acetylsalicylic Acid, 4-Acetoxybenzoic Acid and 5-Acetylsalicylic Acid in Water at 298.15 K

The values of the thermodynamic solubility product, K_sp_, and Δ_s_G^0^ values for acetylsalicylic acid and related compounds were calculated from Equations (6) and (8), respectively, after correcting for ion-pair formation in water.

K^0^_sp_ = a_H_^+^ ∗ a_A_^−^ (6)

In Equation (6), a_H_^+^ and a_A_^−^ represent the activities of the species H^+^ and A^−^ in solution respectively. The activity of the solid is unity by convention. The activity, a, on the molar scale (mol dm^−3^) is related to the ionic molar concentration, c_i_, by the mean molar ionic activity coefficient, γ ± _i_. 

Thus:

K^0^_sp_ = c_i_^2^ ∗ γ ± _i_^2^ (7)

The availability of Gibbs energy (Equation (3)) and enthalpy values allows the calculation of the entropy of solution, Δ_s_S. For this purpose, the following equation was used.

Data are reported in Table 4. Quite clearly, the contributions of the enthalpy and entropy to the Gibbs energy of the process lead to the conclusion that the dissolution of these compounds in water is unfavored.

As far as the enthalpies of solution of acetylsalicylic acid, 4-acetoxybenzoic acid and 5-acetylsalicylic acid in DMF at various concentrations are concerned, the results are reported in Table 5. All observed heats were corrected for the heat from breaking the empty ampoules in DMF (Q = −0.0518 J). Since there were no systematic changes in the Δ_s_H values with changes in the concentration of the solute, the reported standard Δ_s_H^0^ values are the average values of the data reported in Table 5. This table also includes the standard deviations of the data.

Again, the results shown in Table 5 for acetylsalicylic acid and related compounds clearly indicate that their dissolution is endothermic (the enthalpies of solution are positive), and therefore, the contribution of the crystal lattice enthalpy to the Δ_s_H values predominates. Thus, the endothermic character of the reaction follows the sequence:

Acetylsalicylic acid > 5-acetylsalicylic acid > 4-acetoxybenzoic acid

To our knowledge, there are no reports in the literature on the enthalpy of solution of these compounds in DMF.

As no solubility results were obtained for acetylsalicylic acid and related compounds in DMF due to their solvation in this solvent, standard Gibbs energies of solution could not be obtained. Their derivation requires that the composition of these compounds in the solid and in the saturated solution is the same.

As mentioned earlier, Δ_s_H^0^ is made by the contribution of the crystal lattice enthalpy and the solvation enthalpy. In order to remove the contribution of the crystal lattice process, it is a common practice to calculate the transfer thermodynamic parameters according to the following process:

The combination of Δ_s_H^0^ for acetylsalicylic acid and related compounds in water and in DMF, yields the transfer enthalpy, Δ_t_H^0^, of these compounds from the reference solvent to DMF (Equation (8)).

Δ_t_H^0^ = Δ_s_H^0^ (DMF) − Δ_s_H^0^ (H_2_O) (8)

These data provide information regarding the differences in the enthalpic stabilities of these compounds in two solvents and are calculated on the assumption that these compounds are fully dissociated in DMF at infinite dilution. The data reported in Table 6 show that these compounds are enthalpically more stable in DMF than in water.

### 2.5. Interaction of Acetylsalicylic Acid with β-Cyclodextrin in Water and DMF at 298.15 K

The stability constants for the complexation process involving acetylsalicylic acid (nonionized, pH 1.74–1.76, and ionized, pH 5.95–6.09) and β-cyclodextrin in water at 298 K were reported in the literature [15]. In this work, we attempted to obtain the thermodynamic parameters of complexation by titration calorimetry at the above pHs using a four-channel heat conduction microcalorimeter. In both cases, no heats were detected. This was not an indication that complexation does not take place; it simply means that calorimetry is not a suitable reporter of the molecular events taking place, particularly if the process is entropically controlled. It is relevant to mention that there are two different processes taking place at pHs of 1.75 and 6. Thus, at the lower pH the process is defined by Equation (9). As previously discussed by Danil de Namor and coworkers [22], this is referred as an association process, K_ass_:

(9) $$\mathrm{HA}\;(H_{2}O)\;+\;\beta -\mathrm{CD}\;(H_{2}O)\overset{K_{\mathrm{acc}}}{\to }\;\mathrm{HA}\beta -\mathrm{CD}\;(H_{2}O)$$

At pH 6, the process is represented by Equation (10):

(10) $$H^{+}\;(H_{2}O)\;+\;A^{-}\;(H_{2}O)\;+\;\beta -\mathrm{CD}\;(H_{2}O)\overset{K_{c}}{\to }H^{+}(H_{2}O)\;+\;\beta -\mathrm{CD}A^{-}\;(H_{2}O)$$

The thermodynamic data for the processes represented by Equations (9) and (10) are reported in Table 7.

The lower interaction of the ionic relative to the neutral guest with β-CD in water reflected in the K_ass_ and K_s_ values reported in Table 7 must be due to the lower hydrophobic character of the charged relative to the neutral guest, although the higher hydration of the ion in the former relative to the latter is likely to have a negative effect on the penetration of the guest into the hydrophobic cavity of the receptor. Indeed, the more favorable entropy of the neutral relative to the ionic guest is typical for processes in which a higher dehydration or desolvation takes place upon complexation. It is well-established that β-cyclodextrin hosts eight water molecules in its cavity [21], and the higher contribution of the entropy to the more favorable Gibbs energy of the association process provides a clear indication that, in the formation of the adduct, the neutral guest penetrates more into the cavity of the receptor than the ionic guest, and therefore the amount of water released from the cavity is higher in the association than in the complexing process. In both cases, the interaction of the receptor with these guests is entropy-controlled, given that the enthalpic contribution to the Gibbs energy is nil.

As far as the interaction of acetylsalicylic acid with β-cyclodextrin in DMF is concerned, the conductance measurements discussed above showed that, in a dipolar aprotic solvent such as DMF, acetylsalicylic acid is undissociated, and therefore ion pairs predominate in solution. On the other hand the previously reported thermodynamic parameters for the transfer of β-cyclodextrin from water to DMF [21] clearly indicated that this receptor is better solvated in DMF than in water (Δ_t_G^0^ β-CD (H_2_O→DMF)= −4.26 KJmol^−1^). The transfer process is enthalpically controlled, with a high loss in entropy. The most remarkable result found previously was the increase in enthalpic stability with an increase in moving from α to β and to γ cyclodextrin, suggesting that these receptors host the solvent in their cavities. This is in accord with the results shown in Table 7 for the association process of β CD and neutral acetylsalicylic acid in DMF, where the host–guest interaction is greater than that for the same process in water as a result of a more favorable contribution of the entropy resulting from the release of DMF from the ligand cavity. Hence, this release requires energy; therefore, the process is slightly endothermic.

## 3. Materials and Methods

### 3.1. Chemicals

Tris-(hydroxymethyl)aminomethane (THAM) (99%), β-cyclodextrin and molecular sieves (3 Å) were purchased from Aldrich Chemical Company, while N,N-dimethylformamide (DMF) (99.98%) was purchased from Fisher Scientific International Company.

O-acetyl salicylic acid (99%), 4-acetoxybenzoic acid (98%) and 5-acetylsalicylic acid (98%) from Lancaster Synthesis were used in this work.

### 3.2. Solubility Measurements

To determine the solubilities of acetylsalicylic acid (aspirin), 4-acetoxybenzoic acid and 5-acetylsalicylic acid in water and DMF at 298.15 K, saturated solutions of these compounds were obtained by adding an excess amount of the solid to the solvent. The mixtures were left in a thermostatic bath at 298.15 K until equilibrium was reached. Aliquots of the saturated solutions were removed and analyzed gravimetrically by taking a known volume of sample, evaporating the solvent and then weighing the solid in a dry atmosphere. The weighing process was repeated until a constant weight was obtained. The analytical determination was carried out in triplicate on the same equilibrium mixture. Solvate formation was tested by storing the compounds over water or DMF in a desiccator containing an atmosphere saturated with the appropriate solvent over a period of at least four days [38].

### 3.3. Conductance Measurements

The conductivity cell constant was determined by the method of Jones and Bradshaw [39] as previously reported [24]. Conductance measurements were performed using the Wayne Kerr Autobalance Universal Bridge, type B642, and the Wayne Kerr Model 7330 Automatic LCR Meter Conductivity Bridge at a frequency of 1 Hz. Conductometric titrations were carried out in a conductivity cell filled with 50 ml of the solvent (water or DMF) and titrated with a solution of acetylsalicylic acid, 4-acetoxybenzoic acid and 5-acetylsalicylic acid in these solvents (1.00–7.00 × 10^−3^ mol dm^−3^ in water and 1.00 × 10^−2^ mol dm^−3^ in DMF) using a glass syringe connected to a calibrated automatic burette. Conductance readings at 298.15 K were recorded after each addition.

### 3.4. Calorimetric Measurements

Calorimetric measurements were carried out in a Tronac 450, originally designed by Christensen and Izatt [40] for solution studies involving acetylsalicylic acid and related compounds. The reliability of the calorimeter was checked by carrying out the standard reaction based on the protonation of an aqueous solution of tris-(hydroxymethyl)aminomethane (THAM) with an aqueous solution of hydrochloric acid (0.1 mol dm^−3^) at 298.15 K (Equation (11)).

H_2_NC(CH_2_OH)_3_(aq) + H_3_O+ (aq) → H_3_N+C(CH_2_OH)_3_(aq) + H_2_O(aq) (11)

The obtained value (−47.33 kJ mol^−1^) was in good agreement with that of −47.49 kJ mol^−1^ reported by Wilson and Smith at 298.15 K [41]. For deriving the thermodynamic parameters of the interaction of cyclodextrin and these pharmaceuticals in these solvents, the four-channel microcalorimeter was used.

#### 3.4.1. Calorimetric Determination of the Enthalpies of Solution of Acetylsalicylic Acid, 4-Acetoxybenzoic Acid and 5-Acetylsalicylic Acid in Water and DMF

An amount of the solid (acetylsalicylic acid, 4-acetoxybenzoic acid or 5-acetylsalicylic acid) was accurately weighed in a glass ampoule, which was then sealed and placed into the ampoule holder and immersed into the reaction vessel containing the appropriate solvent (50 mL). The system was then placed in a water bath until thermal equilibrium was attained, after which the ampoule was broken into the solvent and the process was monitored by the chart recorder. The observed heat was corrected for the heat of the breaking of the empty ampoule in the solvent. The standard enthalpy of the solution, ∆_s_H^0^, was found from the intercept of a plot of ∆_s_H versus the square root of the concentration (molar scale) of the solution. When no changes in the ∆_s_H were observed with the concentration of the solution, an average value was taken as the ∆_s_H^0^.

#### 3.4.2. Determination of the Thermodynamic Parameters of the Interaction of Acetylsalicylic Acid and β-Cyclodextrin in Water and in DMF

For the calorimetric titrations, a solution of the ligand was placed in the vessel (50 cm^3^) in the selected solvent, and the acetylsalicylic acid was placed in the same solvent in a Hamilton microsyringe. The latter was added from a 2 cm^3^ burette connected by a silicone tube to the reaction vessel after thermal equilibrium was reached. These experiments were carried out in duplicate. For each solvent, blank experiments were performed to account for the dilution effects resulting from the addition of the appropriate anion salt to the solvent placed in the calorimetric vessel.

## 4. Conclusions

This paper highlights the importance of:

(i) Taking into account the speciation in solution when calculating ionic solubility products and therefore the solution thermodynamics associated with these processes;
(ii) Using calorimetry for deriving accurate enthalpy data. The use of the van ’t Hoff isochore leads to inaccurate data due to the limitations of this approach, as clearly stated in the literature;
(iii) Reporting new data in a nonaqueous solvent, such as DMF, which demonstrates the importance of the medium on these systems;
(iv) Assessing the solution behavior of drugs prior to proceeding with binding processes involving macrocyclic receptors as an approach to increase the solubility and therefore the bioavailability of the drug. It is the availability of solution studies of the receptor and the guest that allow the interpretation of the thermodynamics of the interaction of acetylsalicylic acid with the receptor in these solvents. Quite clearly, the two processes taking place in water at different pHs differ from each other, and therefore a different notation should be used to properly identify them.

## Figures and Tables

**Table 1:** Solubilities of acetylsalicylic acid, 4-acetoxybenzoic acid and 5-acetylsalicylic acid in water at 298.15 K.

Compound	Solubility, S (mol dm^−^^3^)
Acetylsalicylic acid	(2.20 ± 0.28) ∗ 10^−2^
4-acetoxybenzoic acid	(6.39 ± 0.01) ∗ 10^−3^
5-acetylsalicylic acid	(3.46 ± 0.22) ∗ 10^−3^

**Table 2:** Λ^0^_m_, K_ip_, K_a_ and pK_a_ values for acetylsalicylic acid, 4-acetoxybenzoic acid and 5-acetylsalicylic acid in water at 298.15 K.

Compound	Λ^0^_m_ (S cm^2^ mol^−1^)	K_ip_	K_a_	pK_a_
acetylsalicylic acid	392	57 × 10^2^	1.75 × 10^−4^	3.76
4-acetoxybenzoic acid	401	22 × 10^3^	4.55 × 10^−5^	4.34
5-acetylsalicylic acid	389	11 × 10^2^	9.09 × 10^−4^	3.04

**Table 3:** Enthalpies of solution of acetylsalicylic acid, 4-acetoxybenzoic acid and 5-acetylsalicylic acid in water at 298.15 K.

Acetylsalicylic Acid		4-Acetoxybenzoic Acid		5-Acetylsalicylic Acid	
c (mol dm^−3^)	Δ_s_H (kJ mol^−1^)	c (mol dm^−3^)	Δ_s_H (kJ mol^−1^)	c (mol dm^−3^)	Δ_s_H (kJ mol^−1^)
2.40 × 10^−4^	11.81	4.51 × 10^−4^	18.9	2.58 × 10^−4^	28.71
3.51 × 10^−4^	10.46	4.57 × 10^−4^	18.33	2.66 × 10^−4^	28.43
4.66 × 10^−4^	10.2	4.66 × 10^−4^	20.25	4.49 × 10^−4^	28.2
5.13 × 10^−4^	11.07	4.90 × 10^−4^	20.51	6.67 × 10^−4^	27.88
5.26 × 10^−4^	11.24	6.17 × 10^−4^	20.62	7.05 × 10^−4^	28.48
5.99 × 10^−4^	11.71	6.75 × 10^−4^	20.99	8.05 × 10^−4^	27.92
Δ_s_H^0^ = 11.1 ± 0.6 kJ mol^−1^		Δ_s_H^0^ = 20 ± 1 kJ mol^−1^		Δ_s_H^0^ = 28.2 ± 0.3 kJ mol^−1^	

**Table 4:** Gibbs free energies, enthalpies and entropies of solution of acetylsalicylic acid, 4-acetoxybenzoic acid and 5-acetylsalicylic acid in water at 298.15 K.

Compound	pK_sp_	Δ_s_G^0^ (kJ mol^−1^)	Δ_s_H^0^ (kJ mol^−1^)	Δ_s_S^0^ (J K^−1^ mol^−1^)
Acetylsalicylic acid	5.45	31.1	11.1	−67
4-acetoxybenzoic acid	6.57	37.5	20	−58
5-acetylsalicylic acid	5.74	32.7	28.2	−15

**Table 5:** Enthalpies of solution of acetylsalicylic acid, 4-acetoxybenzoic acid and 5-acetylsalicylic acid in DMF at 298.15 K.

Acetylsalicylic Acid		4-Acetoxybenzoic Acid		5-Acetylsalicylic Acid	
c (mol dm^−3^)	Δ_s_H (kJ mol^−1^)	c (mol dm^−3^)	Δ_s_H (kJ mol^−1^)	c (mol dm^−3^)	Δ_s_H (kJ mol^−1^)
5.74 × 10^−4^	8.18	1.03 × 10^−3^	3.89	1.11 × 10^−3^	6.62
6.42 × 10^−4^	7.83	1.18 × 10^−3^	4.10	1.45 × 10^−3^	6.65
8.62 × 10^−4^	7.69	1.26 × 10^−3^	3.97	1.76 × 10^−3^	6.55
9.10 × 10^−4^	7.82	1.60 × 10^−3^	3.99	2.07 × 10^−3^	6.66
1.10 × 10^−3^	7.50	1.82 × 10^−3^	3.8	2.18 × 10^−3^	7.07
1.48 × 10^−3^	7.54			2.38 × 10^−3^	6.95
Δ_s_H^0^ =	7.8 ± 0.2 kJ mol^−1^	Δ_s_H^0^ =	3.9 ± 0.1 kJ mol^−1^	Δ_s_H^0^ =	6.8 ±0.2 kJ mol^−1^

**Table 6:** Derived enthalpies of transfer of acetylsalicylic acid, 4-acetoxybenzoic acid and 5-acetylsalicylic acid from water to DMF at 298.15 K.

Compound	Δ_t_H^0^ (kJ mol^−1^)
Acetylsalicylic acid	−3.3
4-acetoxybenzoic acid	−16.1
5-acetylsalicylic acid	−21.4

**Table 7:** Thermodynamic data for the interaction of nonionized (pH 1.75) and ionized acetylsalicylic acid in water and nonionized acetylsalicylic acid in DMF with β- cyclodextrin at 298 K.

WATER				
pHlog K_ass_	log K_ass_	Δ_ass_G^0^ (kJ mol^−1^)	Δ_ass_H^0^ (kJ mol^−1^)	Δ_ass_S^0^ (JK^−1^ mol^−1^)
1.75	2.74 ^a^	−15.64 ^a^	0 ^b^	52 ^b^
**pH**	**log K_s_**	**ΔcG^0^ (kJ mol^−1^)**	**ΔcH^0^ (kJ mol^−1^)**	**ΔcS^0^ (JK^−1^ mol^−1^ )**
it				
6.00	1.71 ^a^	−9.76 ^a^	0^b^	33 ^b^
**DMF**				
**pH**	**log K_ass_**	**Δ_ass_G^0^ (kJ mol^−1^)**	**Δ_ass_H^0^ (kJ mol^−1^)**	**Δ_ass_S^0^ (JK^−1^ mol^−1^ )**
------	4.66 ± 0.03 ^b^	−26.6 ^b^	0.15 ± 0.03 ^b^	89 ^b^

^a^ Calculated from K_ass_ and K_s_ values of 549 and 51, respectively, given in Ref. [15]. ^b^ This work.

## Data Availability

Not applicable.
